# Meningioma Presenting With Intratumoral Hemorrhage on Active Surveillance

**DOI:** 10.7759/cureus.41787

**Published:** 2023-07-12

**Authors:** Rafael Matias, Mavilde Arantes, Júlia Azevedo, Manuel Jacome, Artur Aguiar

**Affiliations:** 1 Radiation Oncology, Instituto Português de Oncologia do Porto Francisco Gentil, Porto, PRT; 2 Neuroradiology, Instituto Português de Oncologia do Porto Francisco Gentil, Porto, PRT; 3 Pathology, Instituto Português de Oncologia do Porto Francisco Gentil, Porto, PRT

**Keywords:** intracranial meningioma, radiotherapy (rt), fractionated stereotactic radiotherapy, radiology report, neuroradiology, intratumoral hemorrhage, meningioma, hemorrhagic meningioma, intracranial radiosurgery

## Abstract

Meningiomas are relatively common primary adult brain tumors. They are slow-growing, highly vascular, and graded according to histology, phenotypic and genotypic features.

We present a case of a 66-year-old male with a history of tongue squamous cell carcinoma, which presented multiple risk factors for cardiovascular and thromboembolic events. A brain lesion was initially detected on a computed tomography (CT) scan and later characterized by magnetic resonance imaging (MRI). The multidisciplinary team decided to maintain surveillance due to the lack of associated symptoms. Upon expansion in size and acute intralesional hemorrhage seen on follow-up imaging, the patient was submitted to surgical excision. The histopathological testing determined it to be an atypical meningioma. Two months later, the patient received stereotactic radiotherapy, and a post-surgical MRI showed no evidence of tumor recurrence.

This case report describes a rare occurrence of intratumoral hemorrhage in a meningioma during surveillance, highlighting the importance of vigilant monitoring and consideration of potential risk factors for hemorrhagic events.

## Introduction

Meningiomas account for 20 to 30% of all primary adult brain tumors. They are slow-growing and highly vascular tumors, commonly found in the supratentorial convexity. They can be classified into different subtypes, each with a specific grade based on the 2016 World Health Organization (WHO) classification. From 2021 according to the new WHO classification, grading will be based on phenotypic and genotypic features. If the following mutations, such as TRET promoter mutation or homozygous deletion of CDKN2A and/or CDKN2B are present, a neoplasm that was previously classified as Grade I would now be a Grade III. A Grade I is considered benign and Grade III malignant according to the 5th WHO classification [1]. Only about 15 to 20% of meningiomas are Grade II and can initially present as benign, but can later become malignant. They often have a gradual onset of symptoms and are usually diagnosed with accompanying headaches, seizures, or other site-specific symptoms [2]. 

Around 4% of all brain tumors will present with spontaneous intracranial hemorrhage, mostly in pituitary adenomas, highly vascularized tumors such as medulloblastoma, neuroblastoma, ependymoma, oligodendroglioma, or metastatic tumors. Hemorrhage is a rare complication of meningiomas, with a reported incidence of 0.5 to 2.4%. Most reported intracranial hemorrhages associated with meningiomas are found in the subarachnoid and subdural spaces. Purely intratumoral hemorrhage is a much rarer event [3-5].

In this report, we present a case of a meningioma presenting with intratumoral hemorrhage on surveillance.

## Case presentation

This is a case of a 66-year-old male followed at an oncologic institution due to a squamous cell carcinoma of the tongue since 2020. The patient had multiple risk factors for a cardiovascular or thromboembolic event, such as heavy smoking habits, diabetes mellitus, dyslipidemia, hypertension, past alcoholic use disorder, and a past embolic event in the right internal jugular vein associated with an implanted central venous catheter used for the treatment of the tongue tumor, managed with anticoagulants.

**Figure 1:**
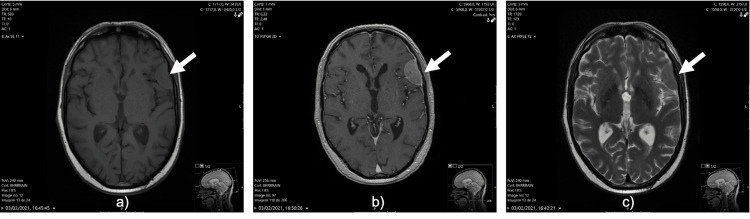
Magnetic resonance imaging showed an extra-axial tumor suggestive of meningioma. (a) Axial T1-weighted images showed a left fronto-opercular, extra-axial lesion with well-defined borders, and isointense to white matter. (b) The lesion showed strong enhancement on post-contrast axial T1-weighted imaging and a dural tail sign. (c) Axial T2-weighted MR images showed that the lesion was slightly hyperintense to white matter; this sequence demonstrated no vasogenic edema in the parenchyma surrounding the lesion.

In January 2021 a computed tomography (CT) of the neck to assess the response of the tongue tumor to neoadjuvant chemotherapy, an intracranial space-occupying lesion was found with no associated symptoms. For a better characterization of the lesion, a brain magnetic resonance imaging (MRI) was performed, which revealed a left fronto-opercular extra-axial lesion with a diameter of 31x20x26 mm and well-defined borders without associated edema (Figure 1). 

**Figure 2:**
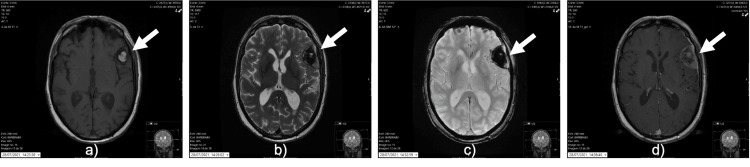
Magnetic resonance imaging at six months of follow-up showed signals of acute intratumoral hemorrhage. (a) Axial T1-weighted images showed areas of hyperintense signal inside the tumoral. (b) Axial T2-weighted MR images showed that the lesion had a heterogeneous signal. The lesion showed a marked loss of signal intensity on T2 gradient-echo images (c) and heterogeneous signal on axial T1-weighted after contrast (d).

It exhibited features that are typical of meningioma. The case was evaluated in a multidisciplinary group, and taking into account the characteristics of the brain lesion and the fact that the patient had no associated symptoms, it was decided to keep close monitoring and perform an imaging control six months later. In the follow-up brain MRI, the lesion showed an increase in size to 36x26x37mm, associated with a heterogeneous signal intensity in all MRI sequences (with hyperintense areas on T1-weighted images and marked loss of signal intensity on T2 gradient-echo images), suggesting intralesional acute hemorrhage. At this time, vasogenic edema was observed along the left frontal lobe causing a mass effect on surrounding structures, which promotes left lateral ventricle compression (Figure 2).

However, no new symptoms or neurologic deficits were associated with the increase in tumor size or the hemorrhagic component. The patient denied any history of a traumatic event.

Due to these new imaging findings, the patient was proposed for tumor removal surgery. The surgery, classified as a Simpson IV, occurred without complications. There were no consequential neurological deficits. 

**Figure 3:**
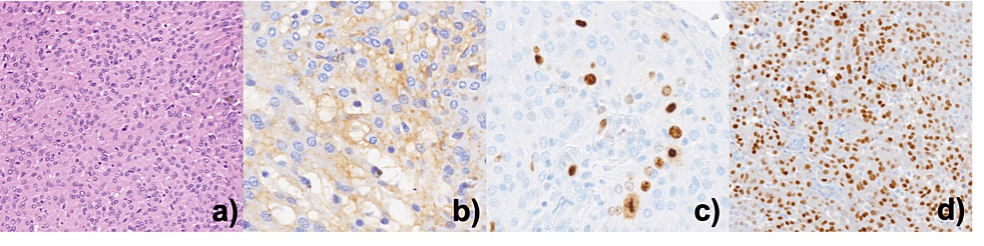
Histopathologic examination. a) Meningeal neoplasia showing high cellularity with indistinct cytoplasmatic borders, nuclei slightly pleomorphic; b) EMA +; c) Ki67 >4%; d) PR +.

Pathologic analysis of the lesion confirmed the histology of the meningioma and classified it as an atypical meningioma (corresponding to a Grade II, according to the WHO classification) (Figure 3). Since the diagnosis was made before the 5th WHO classification, no further genetic studies were performed.

Due to histological features, the patient was submitted two months after surgery to stereotactic radiotherapy, with a total dose of 54 Gy in 27 daily fractions (2Gy/fraction), using volumetric modulated arc therapy (VMAT) with 6MV photons. The predicted tumor volume (PTV) included the tumor bed, possible residual lesion, dural resection bed, and a safety margin. The dosimetric evaluation showed coverage of the target volume (PTV) with D98 of 95%, D95 of 96.6%, and D2 of 104.9%. The maximum dose to the left optic nerve was 51.6Gy, 22Gy to the brainstem, and 19.7Gy to the pituitary gland.

The recovery was uneventful and post-surgical MRI showed no signal of tumor recurrence or growth.

## Discussion

With the advances in imaging diagnostic methods, there has been an increase in incidental findings of brain tumors, namely meningiomas which represent one of the most common benign brain tumors [6]. 

In this case report, we had the opportunity to see, on imaging, signs of intratumoral bleeding in a previously solid mass, emphasizing that the patient remained asymptomatic.

Hemorrhagic events are usually associated with gliomas and metastatic tumors, being rare in meningiomas. The hemorrhagic events associated with meningiomas can be classified as subarachnoid, intracerebral, subdural, or intratumoral hemorrhage. Most bleeding meningiomas often present with extra-tumoral bleeding or even a combination of extra-tumoral and intratumoral bleeding, with only a few cases of exclusively sustained intratumoral meningioma bleeding reported in the literature.

Bosnjak reviewed 145 cases of presumptive preoperative diagnosis of hemorrhagic intracranial meningioma and identified that bleeding propensity is higher in patients older than 70 years and younger than 30 years, and in the fibrous, malignant, and angioblastic histological types of meningiomas [7]. 

The exact mechanism of spontaneous intra-tumoral hemorrhage is not yet understood, but some possibilities have been postulated [8-10]. Usually bleeding events in tumors are related to compensatory hypertrophy of feeding vessels, rapid and abnormal angiogenesis producing friable vessel walls, systemic processes increasing vascular fragility including hypertension and diabetes mellitus, direct vascular invasion by tumor cells, and extensive intratumoral infarction and necrosis [11]. Furthermore, in a recently published case report, other possible risk factors for benign tumor hemorrhagic presentation could be a traumatic brain injury, concomitant anticoagulation, serotonin-modulating therapy, and/or high-dose estrogen replacement [12]. Among the different hypotheses that could explain the bleeding event in our patient, we believe that the blood dyscrasy secondary to anticoagulation medication [13] associated with multiple comorbidities, such as hypertension and diabetes mellitus, together with an abnormal blood vessel architecture, was the cause of the intratumoral hemorrhage. 

## Conclusions

Even though meningiomas rarely present with a hemorrhagic event, this scenario should never be discarded. In our case, the patient had several risk factors for a hemorrhagic event, namely hypertension, diabetes mellitus, and antithrombotic medication, besides the tumor location in the convexity. Although the patient had no symptoms regarding the meningioma, tight control of the lesion was revealed to be of utmost importance in the early identification and fast-acting management and good prognosis of the patient.
